# Risk factors for cutaneous leishmaniasis in a high-altitude forest region of Peru

**DOI:** 10.1186/s41182-021-00332-0

**Published:** 2021-05-17

**Authors:** Justin T. Lana, Andrés Mallipudi, Ernesto J. Ortiz, Jairo H. Arevalo, Alejandro Llanos-Cuentas, William K. Pan

**Affiliations:** 1 Nicholas School of the Environment, Duke University, Durham, NC USA; 2 Duke Global Health Institute, Duke University, Durham, NC USA; 3 The Johns Hopkins University School of Medicine, Baltimore, MD USA; 4 Facultad de Medicina San Fernando, Universidad Nacional de San Martin Tarapoto, Tarapoto, San Martin Perú; 5 Laboratorio Referencial de Salud Publica San Martin, Tarapoto, San Martin Peru; 6 Instituto de Medicina Tropical “Alexander von Humboldt”, Universidad Peruana Cayetano Heredia, Lima, Lima Peru

**Keywords:** American tegumentary leishmaniasis, Agriculture, Coffee, Case control, Deforestation, Urbanization, Neglected tropical disease, Epidemiology, CLASlite, Vector-borne disease

## Abstract

**Background:**

American cutaneous leishmaniasis (CL) is a neglected tropical disease typically associated with men working in remote, sylvatic environments. We sought to identify CL risk factors in a highly deforested region where anecdotal reports suggested an atypical proportion of women and children were infected with CL raising concern among authorities that transmission was shifting towards domestic spaces and population centers.

**Methods:**

We describe the characteristics of CL patients from four participating clinics after digitizing up to 10 years of patient data from each clinic’s CL registries. We assessed risk factors of CL associated with intradomestic, peridomestic, or non-domestic transmission through a matched case-control study with 63 patients who had visited these same clinics for CL (cases) or other medical reasons (controls) between January 2014 and August 2016. The study consisted of an in-home interview of participants by a trained field worker using a standard questionnaire. Risk factors were identified using bivariable and multivariable conditional logistic regression.

**Results:**

Between 2007 and 2016, a total of 529 confirmed CL positives were recorded in the available CL registries. Children and working aged women made up 58.6% of the cases. Our final model suggests that the odds of sleeping in or very near an agricultural field were five times greater in cases than controls (*p* = 0.025). Survey data indicate that women, children, and men have similar propensities to both visit and sleep in or near agricultural fields.

**Conclusions:**

Women and children may be underappreciated as CL risk groups in agriculturally dependent regions. Despite the age-sex breakdown of clinical CL patients and high rates of deforestation occurring in the study area, transmission is mostly occurring outside of the largest population centers. Curbing transmission in non-domestic spaces may be limited to decreasing exposure to sandflies during the evening, nighttime, and early morning hours. Our paper serves as a cautionary tale for those relying solely on the demographic information obtained from clinic-based data to understand basic epidemiological trends of vector-borne infections.

**Supplementary Information:**

The online version contains supplementary material available at 10.1186/s41182-021-00332-0.

## Introduction

American cutaneous leishmaniasis (CL) remains a significant threat in the new world [1–4]. In 2018, the World Health Organization reported over 36,000 cases in South America, three-quarters of which occurred in just three countries: Brazil, Colombia, and Peru [1, 5]. Actual CL case numbers are believed to be much higher than reported figures due to a number of factors, including the lack of healthcare access, poor diagnostics in health clinics, self-treatment or spontaneous healing of skin lesions, and incomplete reporting from health facilities [6, 7]. In Peru, for example, around 7500 CL cases are reported annually [5], though an estimated 17,000 to 30,000 people are believed to develop clinical symptoms each year [1]. CL causes ulcerative, scarring skin lesions that take weeks to months to heal [8].

Over the past 25 years, research from across South America has demonstrated that urbanization and deforestation can alter CL disease dynamics, shifting risk into more settled spaces as wild mammal reservoirs and Phlebotomine sandfly vectors adapt to altered landscapes [9–11]. As a result—given age and gender behavioral and occupational norms—shifts in transmission towards communities and peridomestic spaces can disproportionately increase risk for women, children, and older adults [12]. For example, data from northeast Argentina (elev. 182 m) suggest that individuals living in homes near ponds, woodlands, plantations, secondary vegetation, and agricultural areas have an increased risk for CL, regardless of age or sex [13]. In the Andean foothills of Colombia (elev. 400–1200 m), intradomestic or peridomestic transmission is believed to have occurred in areas where cacao and coffee plantations recently replaced primary forest. Here, the average age of infection among the population was just 7.7 years old. The prospective study found no increased risk between sexes [14]. In northeastern Brazil (elev. 500–800 m), researchers reported a high density of potential sandfly vector species within and near homes; they also found individuals living along urban-rural interfaces in newly developed areas to be at increased risk of developing CL. Once again, the CL burden fell relatively equally on women, children, and men [15]. Though each of these studies represent different human-environment fronts, the authors conclude that CL affects women and children at disproportionate or atypical rates due to domestic or peridomestic transmission.

Considering the limited number of epidemiological CL studies published in recent years [16, 17], much of our understanding of CL risk factors in the Americas is informed by decades old research or on conclusions based on the demographic information of care-seeking CL patients. While each provide great value to our understanding of this neglected disease, neither is sufficient to understand the current epidemiology of CL in a given area, particularly as it relates to shifts in transmission or behavioral risk factors. Thus, we sought to clarify CL risk factors for individuals living in or near typical, small urban areas within an agriculturally dependent, highly deforested, high-altitude forest region of Peru. In this paper, we describe the age-sex spatial distribution of CL cases over 10 years for individuals who accessed one of four CL diagnostic and treatment centers. We report specific CL risk factors associated with intradomestic (within the home), peridomestic (outside but near to homes), and non-domestic (in forested or agricultural areas) transmission obtained from a clinic-based, 1:2 matched case-to-control study for patients visiting these four facilities. And we estimate the population attributable fraction (PAF) attributed to the main risk factor for our study population.

## Methods and materials

### Study area

**Fig. 1:**
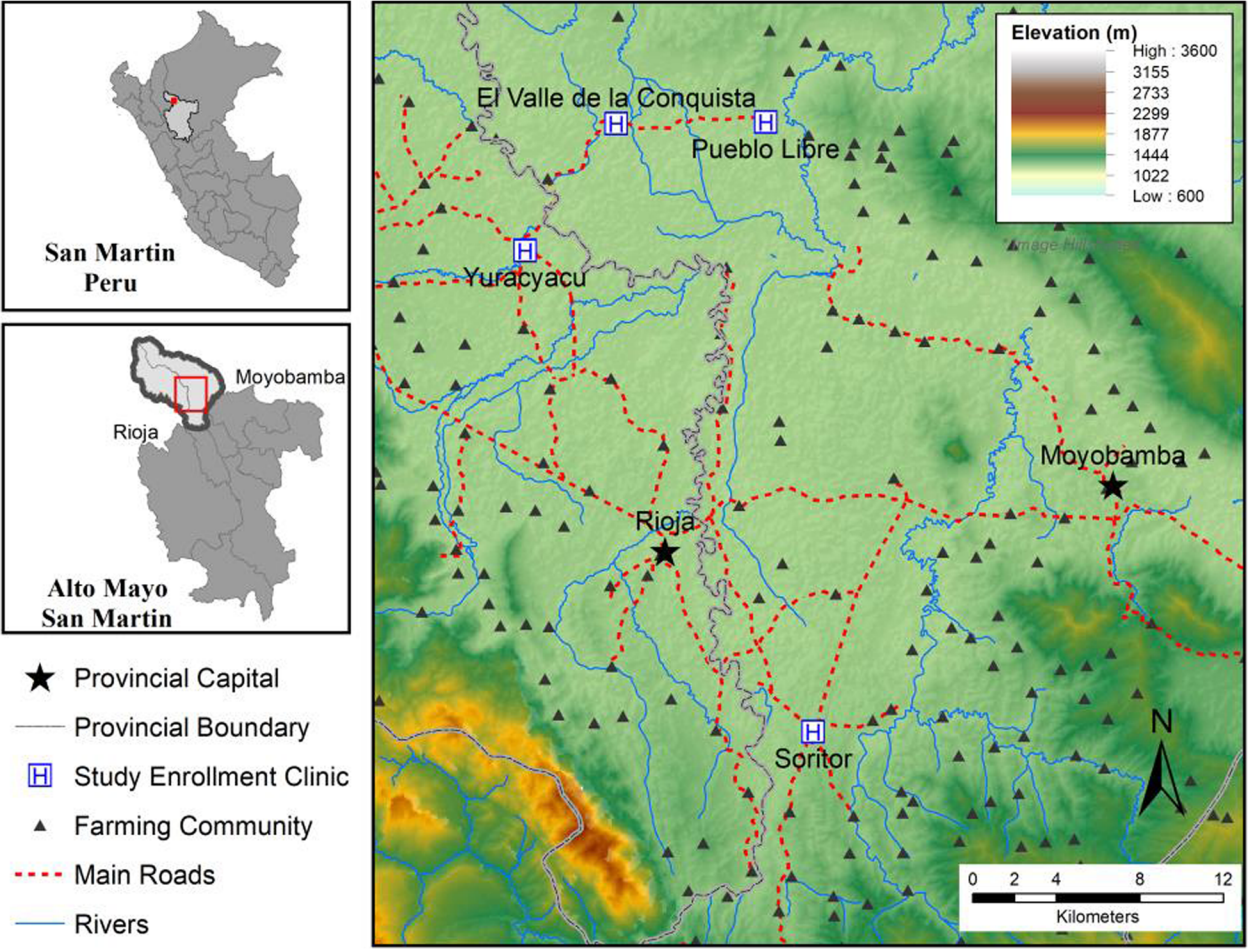
Rioja and Moyobamba provincial regions comprise the area known as the Alto Mayo

This study was conducted outside of the Alto Mayo Protected Forest spanning Moyobamba and Rioja Provinces of the Department of San Martin, Peru (Fig. 1). The Alto Mayo is situated on the eastern slope of the Andes Mountains and is a high altitude Amazon forest zone (elevation 800–1200 m). This region is known for its high production of coffee, cacao, and rice. Due to agricultural expansion, Moyobamba and Rioja Provinces have lost 56,571 ha and 21,509 ha of forest, respectively, from 2001 to 2016 [18].

Our study focused on patients seeking medical care at health centers in four areas: Soritor (population 14,310; elevation 856 m), Yuracyacu (3428; 812 m), Pueblo Libre (2785; 856 m), and El Valle de la Conquista (1676; 799 m). These communities act as commercial centers to the numerous small, rural farming communities scattered across the landscape. The health centers in these communities serve as CL diagnostic and treatment centers in the region; referral centers for smaller, more basic, health posts in select farming communities; and the referring health centers to the larger hospitals in the provincial capitals of Rioja and Moyobamba. Communities were selected in collaboration with the regional health directorate based on anecdotal reports that many women and children were reporting to clinics with CL infections. We note that while each community was classified as urban at the time of study by the Peruvian census bureau [19], the roads remain mostly unpaved; domestic animals, including chicken and pigs, roam freely in the yards or gardens of residents; and small orchards of bananas, papaya, cacao, etc. surround the mostly wooden and corrugated iron homes.

### CL registries and case definition

To assess demographic characteristics of CL patients from the participating clinics, we digitized up to 10 years of data from each clinic’s CL case registries. These registries record basic information from every individual seeking medical care for a suspected CL lesion. Information recorded includes age, sex, date of visit, community of residence, and CL test result. For a patient to be listed as CL positive, a nurse at each facility would have performed a slide smear of each suspected CL lesion or skin ulcer. A trained laboratory technician at each clinic would have then determined the CL test results based on the visualization of *Leishmania* parasites via microscopy of the slide smear.

### Case-control study design

We conducted a 1:2, matched case-control study from July to August 2016. Eligible participants were those who, after January 1st, 2014, had received a positive CL diagnosis (cases) at one of the participating clinics or had visited one of them for other medical reasons (controls). The study consisted of one 30-min in-home interview of study participants by a trained field worker. The standard questionnaire assessed participants’ general demographic information, brief health histories, occupational behaviors, and current living conditions. Accounting for the variable latency period between CL infection and clinical presentation of disease (2 weeks–9 months; mean 8 weeks) [20], field workers conducting interviews instructed all participants to focus their responses to the 9 months of activity prior to their visiting the clinic. Field workers recorded the latitude and longitude of each household following the interview using a handheld global positioning system (GPS) device (accuracy ± 3 m). Participation in the study was voluntary, and all participants (or their guardians) consented prior to enrollment.

### Case and control eligibility

All individuals diagnosed as CL positive via slide smear between January 2014 and August 2016 were screened for participation. Potential controls were identified from these same clinics’ general (non-CL) health registries. Potential controls were defined as individuals presenting to the clinic during the eligibility period with no active CL lesions, no history of past CL (self-reported during interview), and no active skin infections. The last criterion was to prevent patients with undiagnosed CL from becoming controls. We determined that all cases and controls living greater than 6 km from the health center to be ineligible as they, and other residents of their community, typically seek care at small health posts nearer their homes for non-CL-related health needs.

### Matching procedure

Prior to the study’s start, we defined three matching criteria: (1) s*ex*, (2) *age*, and (3) *date of clinic visit*. Collectively, these criteria ensure cases and controls have similar propensities of disease and time periods of recall. *Age* matching was initially defined by age category. For persons <16 years (school age or younger), cases and controls were matched within 3 years of age. For persons 16–65 (working age) and >65 (retirement or old age), cases and controls were matched within 5 years of age. In addition, initial matching by *date of clinic visit* occurred if cases and controls were seen in a clinic within 30 days of each other. However, due to the small number of patients visiting health clinics for non-CL reasons, once enrollment began, we relaxed two criteria to ensure more cases could be paired with at least one control. *Age* criteria were changed to match cases and controls within 10 and 5 years of age for adults and children, respectively, *or* if they fell within the same age categories previously defined. *Date of clinic visit* criteria were changed to ± 100 days (Figure 4, Supplemental). For logistical reasons, cases and their matched controls were recruited from the same clinic. The matched analysis treats this operational decision no differently than our a priori decision to match on *age*, *sex*, or *date of clinic visit*.

### Case and control ascertainment

Once potential participants were identified from the registries, our field team visited their last known addresses to recruit them for participation. Participation was completely voluntary, and no interviews were conducted without having received a signed consent form from participants (or their guardians). If a control could not be located, whenever possible, another potential control was selected from the health registry and sought out for recruitment. Enrollment of cases and controls is summarized in Fig. 3.

### Data analysis

Considering our 1:2 matched design, we required a minimum of 20 matched groups to detect a difference in odds of 4.00 between cases and controls or 30 matched groups to detect a difference in odds of 3.00.

Variables examined as possible CL risk factors were categorized as intradomestic, peridomestic, or non-domestic. Forested areas were identified using CLASlite software [21] with August 2016 Landsat 8 imagery (30-m resolution). Straight line distances between participants’ homes and geospatial variables of interest were obtained using ArcGIS software [22] from shapefiles provided by the local municipalities and the Landsat raster data.

Analyses included bivariable and multivariable conditional logistic regression [23]. Variables marginally important (*p* ≤ 0.10) in the bivariable analysis were included in the multivariable analysis. The final model was fit using forward stepwise regression. We set the model inclusion criteria at *p* ≤ 0.20. Prevalence of exposure in the control group was used to estimate population attributable fractions for the predominant risk factor. Power calculations and analysis were carried out using Stata’s *power mcc*, *clogit*, *stepwise*, and *punafcc* functions [23, 24].

### Ethical approval

The study was reviewed and approved by the Universidad Peruana Cayetano Heredia research ethics committee (SIDISI #66647).

## Results

### Clinic registries

**Fig. 2:**
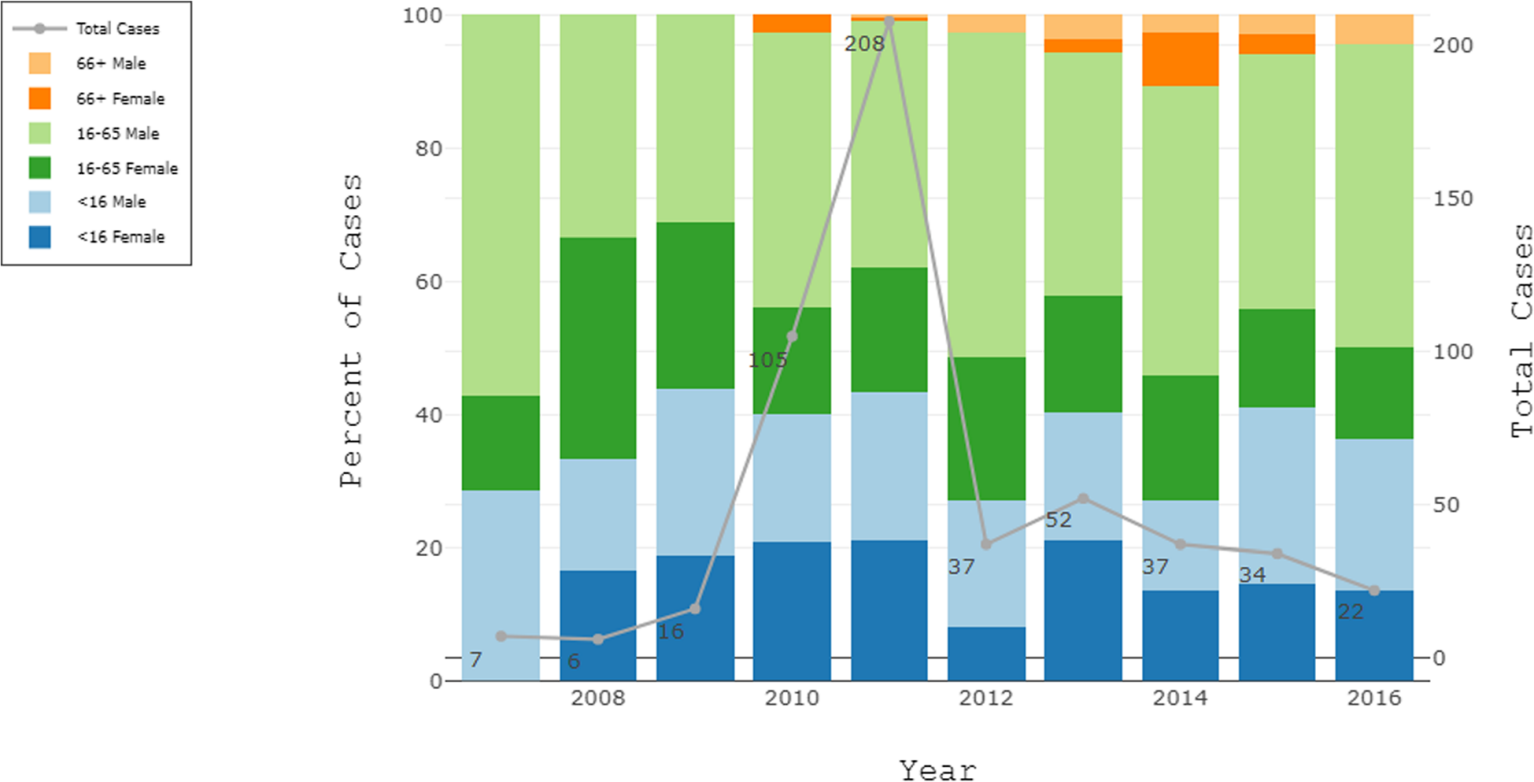
Age-sex distribution of CL cases in four clinics located in Moyobamba and Rioja provinces, Peru, Jan 2007 through July 2016. Data availability: Pueblo Libre, 01/2007–07/2016; Soritor, 01/09–07/2016; Yuracyacu and El Valle de la Conquista (shared registry), 05/2010–07/2016, 2013 missing

Between 2007 and 2016, a total of 529 confirmed CL positives were recorded in the available CL registries of the four participating clinics (Fig. 2). Data availability and completeness varied greatly by year and clinic. Of the recorded data, 39.3% of cases occurred in the <16 age group, 18.1% occurred in women of working age, and 39.5% occurred in working aged men. Only 3.1% of reported cases occurred in the >65 age group. Five males had missing age data. The relative proportion of cases attributed to age-sex groups remained relatively consistent between years despite large differences in the number of cases recorded and available for digitization.

### Case control

**Fig. 3:**
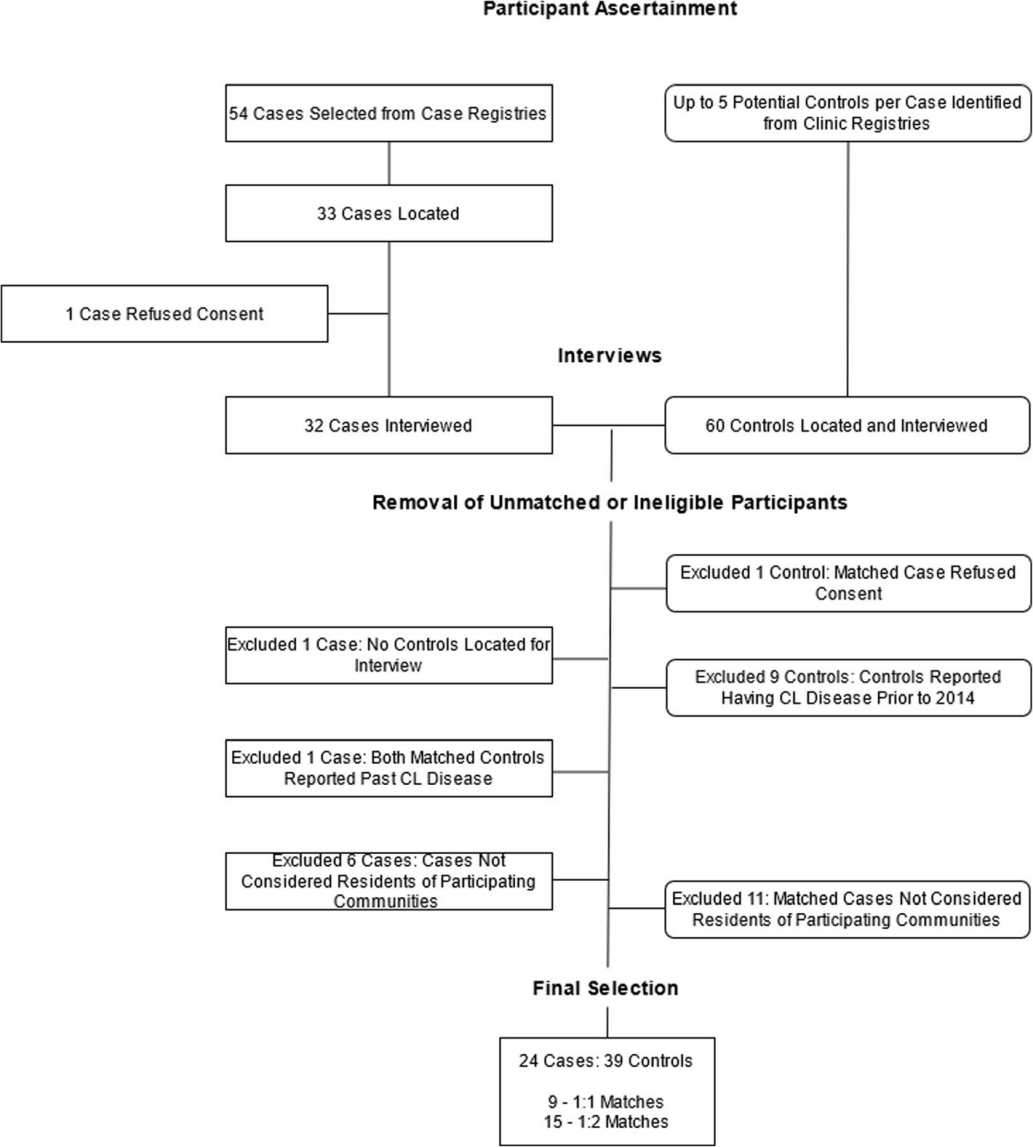
Study enrollment flow chart. Cases and controls identified in the registries were interviewed in no particular order. In some instances, a control was interviewed before their matched case, which explains why controls with no cases may have been interviewed but later dropped from analysis

Of the 93 cases with complete age and sex information listed in the registries beginning January 2014, 39 cases were identified by local nursing staff as ineligible or unavailable for study because they either resided in communities where non-CL-related care was available or they had moved away from the area. Of the 54 cases that remained, we conducted interviews with 32 from July 1st–Aug 20th, 2016. We interviewed 64 controls during this time. We excluded eight cases and 20 controls from the final analysis for reasons outlined in Fig. 3. Final analysis included 24 cases and 39 controls, including nine 1:1 case-to-control matching groups and fifteen 1:2 case-to-control matching groups. Of the cases included in the analysis, 29% were female and 71% were male; 16% of participants were under <16, 16% were over 65, 8% were women of working age, and 58% were men of working age.

### Bivariable analysis

We examined factors associated with intradomestic, peridomestic, and non-domestic CL transmission (Table 1). We found that visiting an agricultural field (OR = 8.79, 95% CI 1.09–70.80, *p* = 0.041), sleeping in or near an agricultural field (OR = 3.844, 95% CI 1.20–12.34, *p* = 0.024), and sleeping in or near a coffee field (OR = 4.41, 95% CI 1.12–16.41, *p* = 0.027) were all behaviors strongly associated with CL infection in the bivariable analysis. Living in a home that was not fumigated during the study period was marginally important (OR = 4.30, 95% CI 0.87–88.74, *p* = 0.073) as was living in a household outside the urban boundary (OR = 2.976, 95% CI 0.885–9.99, *p* = 0.078). No other intradomestic, peridomestic, or non-domestic risk factors were important at an alpha level of 0.10.

**Table 1:** Crude odds ratios (OR) and their 95% confidence intervals (95% CI) for the association between *Leishmania* spp. infection for variables associated with intradomestic, peridomestic, and non-domestic CL transmission, Alto Mayo, Peru 2014–2016

Variables^a^	Cases		Controls		Crude OR	95% CI	*p*-value
	No.	%	No.	%			
**Intradomestic transmission**							
Permanent opening in housing structure							
No	6	25	14	36	1.00		
Yes	18	75	25	64	1.57	0.49–5.02	0.444
Number of rooms							
1 room	5	21	7	18	1.00		
≥ 2 rooms	19	79	32	82	0.81	0.22–2.91	0.742
Type of floor							
Soil or wood	7	29	16	43	1.00		
Cement	17	71	21	57	2.38	0.72–8.01	0.161
Type of wall							
Cement or brick	16	67	33	85	1.00		
Wood	8	33	6	15	2.64	0.75–9.24	0.129
Bednet used last night							
No	1	4	3	8	1.00		
Yes	23	96	36	92	2.18	0.22–21.55	0.503
Living in an unfumigated home							
No	12	52	26	74	1.00		
Yes	11	48	9	26	4.30	0.87–88.74	0.073
Type of roof							
Corrugated iron	22	92	31	79	1.00		
Cement	2	8	8	21	0.40	0.08–21.40	0.073
**Peridomestic transmission**							
Dog ownership							
No	10	42	12	31	1.00		
Yes	14	58	27	69	0.72	0.27–1.91	0.504
Home within 250 m of forest cover							
No	16	76	33	85	1.00		
Yes	5	24	6	15	1.33	0.37–4.73	0.663
Home within 250 m of river							
No	20	95	37	95	1.00		
Yes	1	5	2	5	0.62	0.05–7.00	0.697
Home within 250 m of main road							
No	20	95	35	90	1.00		
Yes	1	5	4	10	0.37	0.04–3.35	0.373
Living outside urban area							
No	12	50	29	74	1.00		
Yes	12	50	10	26	2.98	0.89–9.99	0.078
**Non-domestic transmission**							
Visits an agricultural plot (anytime of day)							
No	1	4	12	31	1.00		
Yes	23	96	27	69	8.79	1.09–70.80	0.041
Visits coffee field (anytime of day)							
No	7	29	18	46	1.00		
Yes	17	71	21	54	2.41	0.69–8.37	0.168
Sleeps in/near a coffee field							
No	14	58	34	87	1.00		
Yes	10	42	5	13	4.41	1.12–16.41	0.027
Sleeps in/near an agricultural plot							
No	13	54	33	85	1.00		
Yes	11	46	6	15	3.84	1.20–12.34	0.024
Exposed to insecticides from agricultural plot							
No	5	23	11	37	1.00		
Yes	17	77	19	63	1.32	0.42–4.42	0.633

^a^ Full data were not available for every subject

### Multivariable analysis

For the multivariable analysis, we conducted a forward stepwise conditional logistic regression with the variables significant or marginally important (*p* ≤ 0.10) in the bivariable analysis: living in an unfumigated home, visiting an agricultural plot, sleeping in or near a coffee field, sleeping in or near an agricultural field, and living outside the urban boundary. Due to the collinearity between those sleeping in an agricultural field and those sleeping in a coffee field, we chose to only include the former in the final model. We found that sleeping in or very near an agricultural field remained significantly associated with CL transmission (*p* = 0.025). Living in a unfumigated home remained marginally important (*p* = 0.062) (Table 2). Living outside the urban area and visiting agricultural fields were eliminated from the final model as they did not meet the established threshold (*p* ≤ 0.20). Population attributable fraction (PAF) estimates indicate that by eliminating the risk of sleeping in or near agricultural plots, 38.3% (95% CI 23.3–50.4%) of CL infections within the study’s catchment area would be prevented. We estimated that 80.1% (95% CI 18.1–95.2) of the infections from the case-control study could be attributed, in part, to this risk factor.

**Table 2:** Adjusted odds ratios (OR) and their 95% confidence intervals (95% CI) for the association between *Leishmania* spp. infection and select variables, Alto Mayo, Peru 2014–2016

Variable	Adj. OR	Adj. 95% CI	*p*-value
Sleeps in/near an agricultural plot	5.04	1.22–20.82	0.025
Living in an unfumigated home	5.35	0.92–31.10	0.062

Our survey results indicate that 25% of females and 28% of males reported sleeping in an agricultural plot, including 27% of those aged < 16, 27% of those of working age, and 28% of those above 65 years old. Furthermore, 79.4% of our sample reported visiting an agricultural area during the 9 months prior to their clinic visit, including all but one case. This individual reported no family ties to agriculture or rural communities and reported no reason to leave her town in the past 2 years. Cases reported 35 CL lesions located on the face (5), upper arm area (4), lower arm area or hands (8), inner thigh (1), and lower leg area or feet (17).

## Discussion

We demonstrated that woman and children living in or near small population centers in the Alto Mayo region of Peru exhibit similar propensities to engage in high risk CL activities as working age men. While the distribution of cases across age and sex reported at the health facility was suggestive of shifting CL risk towards the more populated areas, our matched case-control results indicate that those sleeping in or near agricultural fields are at greatest risk of CL infection. Our conclusion that transmission is most likely to occur away from the urban areas is further supported by the large difference between the total number of cases reported in the case registries and the number of cases reported for individuals living within 6 km of the town centers. This study highlights the importance of looking beyond demographic information obtained from clinic data to define CL risk in an endemic region.

That a larger proportion (62%) of CL infections could not be prevented by eliminating the risks associated with sleeping in or near agricultural plots (as our PAF estimates suggest) signals that other behaviors may be contributing to or mitigating the CL burden in our study area. Over three-quarters of participants reported visiting the agricultural areas, whereas about one-quarter reported staying overnight. Considering sandflies typically bite between 19:00 and 6:00, infections may also be occurring outside of typical sleeping hours [25, 26]. Thus, efforts to reduce infections should go beyond increasing bednet usage and improving sleeping structures for those in the fields; they should encompass education campaigns that encourage *all* individuals to keep exposed skin covered while in agricultural settings especially in the evening, nighttime, and early-morning hours. The high percentage of cases (96%) and controls (92%) who slept under a bednet the night before their interview is notable. Uniformly high bednet usage would reduce transmission while masking some of the effect of fumigation if intradomestic transmission was occurring. Given the marginal importance of living in an unfumigated home in our bivariable and multivariable analyses—combined with what we learned from our interviews—we cannot rule out that transmission might occur in or near any of our participating communities, though we believe urban transmission would represent only a small fraction of all transmission and would most likely occur at the urban periphery.

To the best of our knowledge, this is the first study of CL risk factors from a high-altitude forest region in Peru [16]. Our findings share similarities with other studies from Latin America. In the mid-1990s, Llanos and Davies’ work from the Andean Highlands found that CL increased risk in those sleeping in temporary crop shelters and performing nighttime agricultural activities like crop irrigation [11, 27]. More generally, numerous studies have found associations between CL and coffee and cacao farming [28–32]. Our study results differed from studies described in the introduction that found evidence of intradomestic or peridomestic transmission in highly deforested zones where women and children made up a plurality of cases. Our results underscore the need for more regular epidemiological study to define risk behaviors and monitor changes over time. Even in areas where CL disproportionately affects working aged men, analyses stratified by age or sex may be necessary to better understand shared risk factors across population groups. Classification and regression tree (CART) analyses could help define risk profiles for those not belonging to the predominant risk group.

Our study had limitations. The study occurred in a time of when few cases were reporting to the clinic which limited our sample size. It is unclear if the low case counts were due to low transmission or low treatment-seeking behavior. Because of our clinic-based design, our results do not inform us about populations that do not seek out, lack access to, or have distrust of public health care providers. The study also took place in an area with high emigration, likely biasing our sample towards less migratory individuals. As a result of the limited sample size, the confidence intervals of the variables we examined were large. This limited our ability to draw stronger conclusions about risk factors or to differentiate between risk factors with more precision. While our matched study design allowed us to examine risk factors beyond age and sex, by performing a matched analysis, we were unable to explain, statistically, how age and sex interact with the other covariates of interest. Furthermore, other endemic areas have reported that up to 30% of those exposed to *Leishmania* do not develop infection [14, 33–35] and that up to 50% of those tested for CL are falsely determined to be negative [36, 37]. Thus, our pool of potential cases may have represented a population more prone to develop CL infections or whose infections were more severe; our pool of controls may have included individuals with past exposure that were never diagnosed or never developed symptoms. Inclusion of any non-susceptible individuals as controls would have biased our results towards the null. It remains unknown what percentage of the population in this area would have some level of acquired immunity to CL due to past infection, how the risk factors for infection differ in the more rural communities, or even which species of *Leishmania* exist and are causing infections in the region.

## Conclusions

Despite the limitations, it is clear that women and children may be underappreciated as a CL risk group, especially in areas with agriculturally dependent economies. Curbing transmission in non-domestic spaces may be limited to decreasing exposure to sandflies in agricultural areas. If deforestation and agricultural expansion continues, these small, but growing, population centers may see increased case numbers in the coming years as a result of CL transmission shifting towards domesticated spaces; economic forces driving the activities of men, women, and children into non-domestic spaces; or both.

## Supplementary Information

**Additional file 1: Supplemental Figure 4.** *Age* and *date of clinic visit *differences between cases (0,0 on graph) and their matched-controls (depicted as described in legend).

## Data Availability

The datasets generated and/or analyzed during the current study are not publicly available because they contain identifying information for patients seeking care at a health facility. They are available from the corresponding author on reasonable request and approval of a new IRB.

## Abbreviations

CL: American cutaneous leishmaniasis
PAF: Population attributable fraction
OR: Odds ratio
CART: Classification and regression tree
GPS: Global positioning system
