# Supplementary material for: Risk factors for cutaneous leishmaniasis in a high-altitude forest region of Peru
Source: Trop Med Health. 2021 May 17;49:40. doi: 10.1186/s41182-021-00332-0 (PMC8130303; doi:10.1186/s41182-021-00332-0)
Supplement: Supplementary file 1 — Additional file 1: Supplemental Figure 4. Age and date of clinic visit differences between cases (0,0 on graph) and their matched-controls (depicted as described in legend). [file 41182_2021_332_MOESM1_ESM.docx]

**Supplemental**


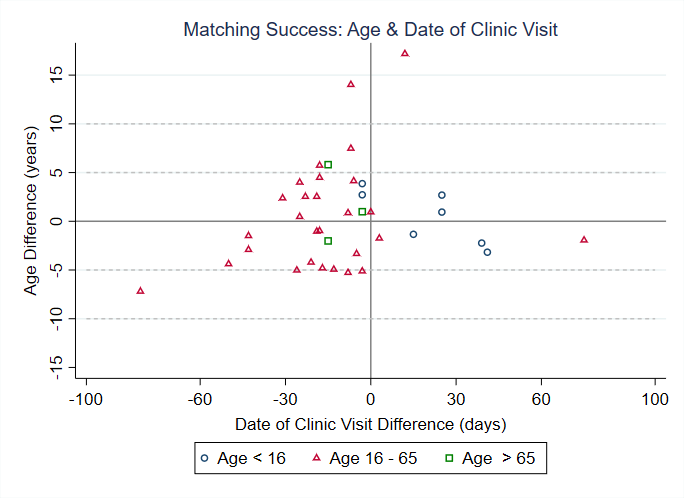


Figure 4: *Age* and *Date of Clinic Visit* differences between cases (0,0 on graph) with their matched-controls (depicted as described in legend).
